# The Improvement of Durability of Reinforced Concretes for Sustainable Structures: A Review on Different Approaches

**DOI:** 10.3390/ma15082728

**Published:** 2022-04-07

**Authors:** Luigi Coppola, Silvia Beretta, Maria Chiara Bignozzi, Fabio Bolzoni, Andrea Brenna, Marina Cabrini, Sebastiano Candamano, Domenico Caputo, Maddalena Carsana, Raffaele Cioffi, Denny Coffetti, Francesco Colangelo, Fortunato Crea, Sabino De Gisi, Maria Vittoria Diamanti, Claudio Ferone, Patrizia Frontera, Matteo Maria Gastaldi, Claudia Labianca, Federica Lollini, Sergio Lorenzi, Stefania Manzi, Milena Marroccoli, Michele Notarnicola, Marco Ormellese, Tommaso Pastore, MariaPia Pedeferri, Andrea Petrella, Elena Redaelli, Giuseppina Roviello, Antonio Telesca, Francesco Todaro

**Affiliations:** 1 Department of Engineering and Applied Sciences, Università di Bergamo, Viale Marconi 5, 24044 Dalmine, Italy; marina.cabrini@unibg.it (M.C.); denny.coffetti@unibg.it (D.C.); sergio.lorenzi@unibg.it (S.L.); tommaso.pastore@unibg.it (T.P.); 2 Department of Chemistry, Materials and Chemical Engineering “G. Natta”, Politecnico di Milano, Via Mancinelli 7, 20131 Milan, Italy; silvia.beretta@polimi.it (S.B.); fabio.bolzoni@polimi.it (F.B.); andrea.brenna@polimi.it (A.B.); maddalena.carsana@polimi.it (M.C.); mariavittoria.diamanti@polimi.it (M.V.D.); matteo.gastaldi@polimi.it (M.M.G.); federica.lollini@polimi.it (F.L.); marco.ormellese@polimi.it (M.O.); mariapia.pedeferri@polimi.it (M.P.); elena.redaelli@polimi.it (E.R.); 3 Department of Civil, Chemical, Environmental and Materials Engineering, Università di Bologna, Via Terracini 28, 40131 Bologna, Italy; maria.bignozzi@unibo.it (M.C.B.); stefania.manzi4@unibo.it (S.M.); 4 Department of Mechanical, Energy and Management Engineering, Università della Calabria, Via Bucci-Cubo 46C, 87036 Rende, Italy; sebastiano.candamano@unical.it (S.C.); fortunato.crea@unical.it (F.C.); 5 Department of Chemical, Materials and Industrial Engineering, Università “Federico II” di Napoli, Piazzale Tecchio 80, 80125 Naples, Italy; domenico.caputo@unina.it; 6 Department of Engineering, Università Parthenope di Napoli, Via Amm. Acton 38, 80133 Naples, Italy; raffaele.cioffi@uniparthenope.it (R.C.); francesco.colangelo@uniparthenope.it (F.C.); claudio.ferone@uniparthenope.it (C.F.); giuseppina.roviello@uniparthenope.it (G.R.); 7 Department of Civil, Environmental, Land, Building Engineering and Chemistry, Politecnico di Bari, Via Orabona 4, 70126 Bari, Italy; sabino.degisi@poliba.it (S.D.G.); claudia.labianca@poliba.it (C.L.); michele.notarnicola@poliba.it (M.N.); andrea.petrella@poliba.it (A.P.); francesco.todaro@poliba.it (F.T.); 8 Department of Civil Engineering, Energy, Environmental and Materials, Università Mediterranea di Reggio Calabria, Via dell’Università 25, 89122 Reggio Calabria, Italy; patrizia.frontera@unirc.it; 9 School of Engineering, Università della Basilicata, Viale dell’Ateneo Lucano 10, 85100 Potenza, Italy; milena.marroccoli@unibas.it (M.M.); antonio.telesca@unibas.it (A.T.)

**Keywords:** concrete durability, rebars corrosion, design strategies, alternative binders

## Abstract

The topic of sustainability of reinforced concrete structures is strictly related with their durability in aggressive environments. In particular, at equal environmental impact, the higher the durability of construction materials, the higher the sustainability. The present review deals with the possible strategies aimed at producing sustainable and durable reinforced concrete structures in different environments. It focuses on the design methodologies as well as the use of unconventional corrosion-resistant reinforcements, alternative binders to Portland cement, and innovative or traditional solutions for reinforced concrete protection and prevention against rebars corrosion such as corrosion inhibitors, coatings, self-healing techniques, and waterproofing aggregates. Analysis of the scientific literature highlights that there is no preferential way for the production of “green” concrete but that the sustainability of the building materials can only be achieved by implementing simultaneous multiple strategies aimed at reducing environmental impact and improving both durability and performances.

## 1. Introduction

In the field of construction materials, it is increasingly evident that traditional environmental parameters (such as global warming potential (GWP), and gross energy requirement (GER)) as well as life cycles analyses are needed—but not sufficient—to define the sustainability of a building material. Simple parameters based on concrete composition, CO_2_ emissions, and compressive strength such as those proposed by Damineli et al. [1] are no longer adequate for a holistic treatment of the issue. It is essential to combine information regarding the material performances and durability with the evaluation of its environmental impact. In other words, it is not possible to define a construction material as “green” without a deep investigation of its property evolution in different environments over time.

The phenomena of early degradation, primarily those promoted by carbon dioxide or chlorides, can greatly reduce the sustainability of cementitious materials, both traditional and innovative, as widely reported in the scientific literature [2,3]. Therefore, this review aims to collect the main strategies currently available for obtaining durable and sustainable reinforced concrete structures, using both traditional and innovative materials.

## 2. Corrosion Mechanisms in Reinforced Concrete Structures

The protective capacity of reinforced concrete against carbon steel corrosion is one of the fundamental points that have made it the most used construction material for industrial and civil structures. Steel reinforcements give tensile strength to cementitious materials, and concrete offers protective conditions to preserve the steel from corrosion, thus making production of durable structures possible. The protective action is due to the formation of hydration products of Portland cement, which increases the alkalinity of the water inside the pores of the hardened concrete. In fact, the corrosion behavior of carbon steel is strongly influenced by the pH of the pore solution, and it is assumed that it is passive when it exceeds 11.5. In these conditions, the corrosion rate of carbon steel reinforcements becomes negligible due to the formation of a protective passive film, which slows down the anodic process of metal dissolution. Portland cement is composed by calcium silicates, which, reacting with water during the hardening process, lead to the formation of calcium hydroxide. This substance is a strong, slightly soluble hydroxide, which saturates the water of the pores. At room temperature, a simple saturated solution of this substance has a pH around 12.5. However, the pH of fresh cement paste is generally higher due to the presence of small amounts of sodium and potassium hydroxides, determining the increase in the pH up to 13.5. These alkalinity levels are reached immediately during the mixing, thus promoting a rapid passivation of the reinforcement [4,5]. The free corrosion potential of rebars rapidly increases, during the setting and hardening phase, up to potentials typical of passive conditions [6,7]. Fresh concrete is a suspension of water, solid particles of different granulometry, and cement dust, where water represents an amount of only about 20%. The solution in contact with steel reinforcements is limited to the adjacent water film, while the solid/liquid ratio increases as the degree of hydration increases. The alkali content of this water thin layer, responsible for the passivity of steel, does not depend only on the content of the above-mentioned hydroxides or on the possible presence of pozzolanic material, but also on the consumption of hydroxyl ions for the formation of the passive film itself. The protectiveness tends to increase over time and it becomes stable only after several months embedded in the cement matrix, as reported also by Andrade et al. [8].

The protective action by Portland cement concrete, however, is not only due to high pH values, but it also depends on the presence of chlorides and on the ability of the cement matrix to decrease the chloride and carbonation penetration through the concrete cover.

Chlorides break the passive film and promotes localized corrosion initiation of reinforcements. This is the main form of corrosion responsible for damaging concrete exposed in the marine environment or bridge decks and civil buildings exposed to de-icing salts. Localized corrosion initiation occurs once chloride concentration (by percentage to the weight of cement) exceeds a critical concentration threshold at the steel surface. In structures exposed to the atmosphere, where the embedded steel rebars are characterized by a high corrosion potential, this critical threshold in Portland cement concrete is usually between 0.4 and 1% [9]. Higher values are found in water-saturated concrete, in which the steel corrosion potential is lower. The alkalinity and characteristics of the concrete/reinforcement interface are the main factors influencing the critical chloride concentration threshold [10,11,12,13,14,15,16,17,18,19]. It increases with the pH and it can be described in terms of chloride to hydroxyl critical molar ratio, which is commonly considered equal to 0.6. According to this ratio, the alkalinity of Portland cement concrete therefore makes possible localized corrosion initiation only when chlorides penetrate from the environment. The value can be even higher in concrete, due to the buffering effect produced by calcium hydroxide formed during hydration of cement [12,20]. The presence of this phase on the metal surface represents a “reserve of alkalinity”, which contrasts, at the metal/cement paste interface, the pH variations involved in the initiation mechanism of localized corrosion. Only a fraction of the total chlorides already present in concrete contributes to the initiation of localized corrosion. Free chlorides, dissolved in the solution contained in the pores, are active, while a significant part is bound by the constituents of the cement [21] and does not influence the corrosion phenomenon. The two main bonding mechanisms of chlorides are by adsorption, especially on hydrated calcium silicate (C-S-H) [22], and by chemical substitution, in monosulfate calcium aluminate (phase AFm) [23,24,25], with the formation of Friedel’s salt. In addition to these phases, chlorides can also adsorb on portlandite (CH), ettringite (AFt), and other salts [25,26,27,28].

The durability of reinforced concrete structures is strictly related to the two main processes governing the corrosion of steel reinforcements, such as chlorides penetration and carbonation. Both processes affect the protective ability of concrete against steel rebar corrosion. The chloride and carbon dioxide penetration rates are mainly dependent upon the porosity of the concrete matrix, the size and distribution of the pores. It is well known, in fact, that the durability of concrete mainly depends on the mix design, placing and curing. In this view, the concrete cover thickness can be considered as the key determining factor which defines the time required for aggressive substances to reach the reinforcements.

The low corrosion rate of the reinforcements is determined mainly by passivity. Oxygen is normally present and reaches the surface of the reinforcement in amounts that promote the corrosion process. Once the passivation layer is broken, however, very different corrosion conditions can occur, in relation to the water saturation of pores. Only in water-saturated concrete, the reduced supply of oxygen, due to the slow diffusion through the pores occluded by the aqueous phase, can limit the corrosion process. This can be observed in permanently immersed concrete, in which even the possible loss of passivity would not lead to any significant corrosion [29]. However, the concrete is not typically saturated with water and the access of oxygen is such as not to constitute a limiting factor, due to the rapid diffusion through the air contained in the pores, only partially filled with water. In this case, the corrosion rate is determined by the availability of water, necessary to promote the corrosion process.

In very humid, but not saturated, concretes, the corrosion process can take place with significant rates mainly in the presence of significant chloride contamination. In these concretes, the amount of water is enough to guarantee a low electrical resistivity of the cementitious matrix, thus favoring the galvanic couple action, which controls the localized corrosion mechanism.

In carbonated concrete—without chlorides—the corrosion rate is much lower and general corrosion occurs. The corrosion rate assumes relatively low values, especially in concretes exposed to low humidity levels. The amount of electrolyte is very low, and consequently the corrosion rate is also low. In addition, the corrosion products tend to reduce the small volume of electrolyte, thus promoting the formation of patinas on reinforcements, which further decrease the anodic oxidation process of the metal. A situation of pseudo-passivity arises, with relatively high corrosion potentials, but with negligible corrosion rates. The propagation period becomes the main process in the service life of carbonated structures.

In addition, the presence of cracks and defects could represent a preferential access point for corrosive agents in concrete. However, Portland cement concrete has “smart” properties that hinder this effect, making it much less important than might be expected. The interaction between concrete and the environment leads to the precipitation of substances that tend to seal the cracks, thus making them much less critical. This is what happens, for example, in the marine environment or in contact with water that contains bicarbonate ions, calcium ions and magnesium ions, in the form of dissolved salts. In contact with the alkalinity of the concrete walls, calcium and magnesium carbonates limit the ingress of water, and they can seal relatively large cracks (below 300 μm) [30]. The barrier properties of concrete are somehow restored, thus prolonging the initiation of the corrosion phenomena. The effect is significant only for small-sized cracks and depends on the characteristics of the water and the properties of the concrete [31,32]. In the presence of major defects, however, this effect cannot be considered, and corrosion rate mechanisms are that of atmospheric corrosion rather than that of corrosion of carbon steel reinforcements in concrete.

## 3. Corrosion Inhibitors and Surface Treatments

Additional protection methods are necessary for reinforced concrete structures operating in severe field conditions or when very long service life is required: corrosion-resistant reinforcements, cathodic prevention, corrosion inhibitors, and surface treatments represent suitable “tools” to prevent corrosion in very aggressive environments [33].

Surface treatments to apply on the surface of reinforced concrete elements are efficient protective methods at a relatively low cost. The European Standard EN 1504-2 identifies:

(a) Hydrophobic treatments, based on silanes, siloxanes and silicones;
(b) Treatments able to seal the capillary pores, based on sodium silicate or magnesium fluorosilicates;
(c) Organic coatings forming a continuous film, with a thickness between 0.1–0.3 mm, thermoplastic (acrylic, vinyl) or thermosetting (epoxy, polyurethane);
(d) Cementitious mortars containing acrylic or vinyl polymers with polymer/cement ratio in the range of 0.3–0.6 and thickness between 1 and 5 mm.

The effect of these treatments is for two reasons: they reduce the transport of aggressive agents in concrete (oxygen, carbon dioxide, and chlorides), delaying corrosion initiation; they decrease the concrete water content, reducing the corrosion rate.

Many laboratory tests have been carried out to study their effectiveness, even if they are mainly short-term tests on water absorption, vapor permeability, adhesion, and accelerated chloride corrosion [34,35,36,37]. Hydrophobic treatment and polymer modified mortars showed the best efficiency on corrosion prevention. A long-term chloride corrosion test, lasting 17 years, showed that polymer modified coatings both delay the initiation of chloride corrosion, thanks to a strong decrease in the chloride penetration, and reduce corrosion rate [38,39]. The higher the polymer/cement ratio, the higher the coating effectiveness. However, few field-tests are available to predict the durability of surface treatments beyond a period of more than 10 years under different conditions of exposure [33].

Corrosion inhibitors can be used to both prevent and stop chloride induced corrosion and as a remedial for structures exposed to carbonation. They can be divided in two groups: admixed inhibitors (mass inhibitors), directly added as a constituent to fresh concrete, and as preventive techniques; migrating inhibitors, applied on the concrete surface which can penetrate into the hardened cement matrix, usually adopted in rehabilitation [40,41,42,43,44]. Among the mass inhibitors, inorganic ones were firstly studied since the 1950s and efficient commercial products are available. Migrating commercial corrosion inhibitors were proposed in the last 30 years, due to the growing interest in the recovery and restoration of existing buildings.

Nitrite-based inhibitors [40,45], acting as anodic passivating agents, are the most effective ones, provided a chloride/nitrite molar ratio lower than 1 is maintained. In the maximum dosage (30 L/m^3^) they guarantee an increase in the critical chloride content up to 3% by cement mass. They also have an effect on carbonation corrosion if dosed at 3% by cement mass. In particular, nitrites were found effective in accelerating the passivation process of active galvanized steel in fresh concrete, which is a significant aspect to consider for these types of reinforcements [46]. Concerns are with its harmfulness, solubility, and possible increase in corrosion rate in the case of low dosage.

Organic commercial inhibitors (amines, alkanolamines, and carboxylates) [40,47,48] act by adsorption on the metal surface, forming an organic monolayer. Laboratory tests, both in solution and in concrete, showed a slight increase in the critical chloride content (up to 1.2–1.5% by cement mass) for inhibitor dosages ranging from 1.5 to 10 L/m^3^. Few data are available on long-term efficiency; in any case, they are not as efficient as nitrite. Migrating organic inhibitors, based on similar compounds, in most cases do not reduce corrosion rate after initiation, they only delay the initiation of corrosion due to a pore blocking effect [40,49].

In the last 20 years there has been a growing interest in the study of new compounds, and to understand the mechanism of inhibition: both inorganic (zinc oxide, molybdates, borates) and organic compounds (benzoate derivatives, carboxylated ions, and amine-based substances) have been tested [13,50,51].

## 4. Self-Healing Strategies for High Durability Concrete

Concrete is a low-tensile strength and fragile material that is very susceptible to cracking mainly due to shrinkage, tensile stress, and freezing and thawing cycles. Generally, microcracks do not significantly jeopardize the elastomechanical performance of concrete but promote an easier penetration of external matters such as water and other chemical agents (i.e., sulfates, chloride, and acids) resulting in cement matrix degradation followed by a corrosion of steel rebars [52,53,54]. In other words, the microcrack formation is generally responsible for a reduction in a service life of concrete structures without affecting their strength [55]. For this reason, the development of techniques aiming at increasing the lifespan or reducing the maintenance costs of buildings are essential, especially in a sustainable perspective of concrete structures [56,57]. In the last years, starting from the autogenous self-healing phenomena described by Hyde and Smith [58] and Glanville [59], researchers investigated several self-healing approaches able to improve the natural capability of concrete to fill cracks.

The autogenous self-healing is defined as the natural recovery process of concretes not specifically designed for self-healing [60] and it occurs due to physical, chemical, and mechanical phenomena. The physical cause is due to swelling of hydrated cement paste next to the cracks, whereas the chemical processes are related to the continued hydration of cement and the formation of calcium carbonate crystals on the crack’s faces. Minor effects are due to mechanical causes such as the presence of fine particles that partially fill the cracks. However, the effectiveness of autogenous self-healing is rather limited and affects only the small cracks with width lower than 300 µm [61,62].

**Figure 1:**
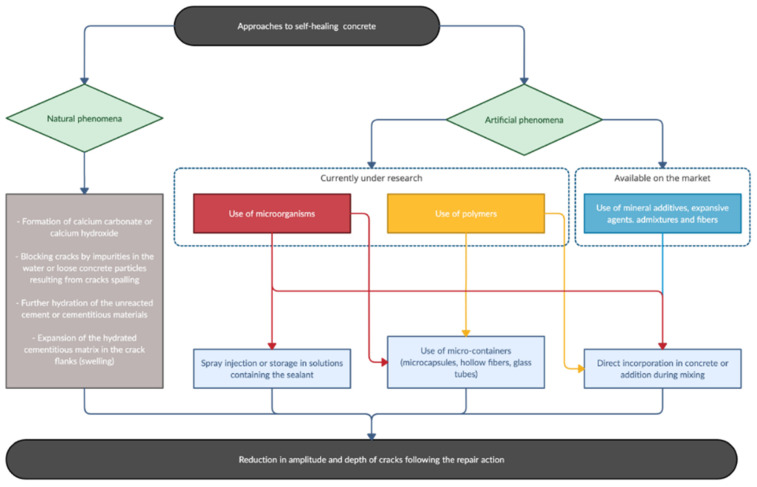
Different approaches to self-healing.

When concrete is manufactured with engineered additions able to improve the self-healing capability of mixtures, it is called autonomic self-healing or activated repairing. Several techniques have been proposed in this field, as reported in Figure 1.

The use of bacteria (also called bacterial concreting) has been shown to be effective in repairing cracks in concrete, promoting both a reduction in water penetration and chloride ion permeability with small recovery in mechanical strength [63,64]. In particular, the microbially induced calcium carbonate precipitation can occur by adding bacteria in porous aggregates [65,66,67], diatomaceous earth [68], rubber particles [69], plastic microcapsules [70,71,72], or hydrogel [73]. In any case, the effectiveness of long-term self-healing capability remains to be assessed.

A technique similar to the bacterial concreting involves the use of polymeric-based repairing agents (such as epoxy resin, methyl-methacrylate, ethyl-cyanoacrylate, or polyurethane) stored in hollow glass fibers [74], ceramic tubes [75], porous plastic fibers [76], or micro-/macrocapsules [77,78]. The healing ability is related to the microcapsule damage around the cracks that releases the healing agent. Inorganic agents (sodium and potassium silicate) were also successfully investigated in [79,80]. Nevertheless, issues related to rheology, dispersion of microcapsules, and mechanical strength loss must be solved before a widespread use of these systems [60].

Crack healing capability of concrete can be also be enhanced by adding in the mixture cross-linked polymers (also called superabsorbent polymers) that have the ability to absorb huge amount of water from the environment and to retain the liquid within their structure without dissolving. When cracks occur, these materials are exposed to the external environment and the subsequent contact with water or moisture promotes the swelling of polymers and the formation of a soft gel that prevents the ingress of external agents into concrete [81,82]. The detailed healing mechanism of superabsorbent polymers has been reported by Lee et al. [83].

The most promising technique for autonomic self-healing is the addition of expansive agents, mineral additives (also called supplementary cementitious materials), admixtures and fibers as well as their combination during the mixing [84,85,86]. Several studies evidenced that the addition of expansive agents (i.e., MgO, CaO, bentonite) and fibers both limits the shrinkage of concrete and produces compatible expansive hydrated. In this way, crack bridging capacity (strength recovery), sealing (physical closer of cracks through crystallization), and durability are improved [87,88]. On the other hand, the use of mineral additives and carboxylic acid derivatives promotes both the cement recrystallization and the salt precipitation inside cracks with an initial width up to 500–800 µm without affecting the properties of concrete at fresh and hardened state [89,90]. More details can be found in [30].

## 5. Corrosion Resistant Reinforcements

When the concrete cover is not able to provide the proper protection against corrosion of the traditional carbon steel reinforcement, e.g., in highly aggressive environmental conditions (especially in the presence of chlorides) or when a long service life is required, it is possible to use additional prevention/protection systems in order to guarantee the required durability [91]. The use of corrosion resistant reinforcements is one of the main additional prevention/protection systems and can be a sound choice for new structures or in repair of existing ones. The corrosion resistance of reinforcements can be obtained with coatings, both metallic (galvanized steel) or organic (epoxy coated bars), modifying the chemical composition of the steel (mainly using stainless steels) or using composite materials (FRP, Fiber Reinforced Polymers) [33,92].

The corrosion resistant reinforcement should fulfil the requirements settled for the traditional carbon steel bars, such as strength, ductility, weldability, and bond to concrete. These rebars are characterized by different corrosion behavior and costs. Their related benefits can be evaluated with performance-based approaches for the design of durability [93]. As far as the costs are concerned, although their higher initial costs, their use can lead to significant costs savings during the service life of the structure, due to a reduction in maintenance costs (direct and indirect). Moreover, a selective use in the most critical parts can be considered, thus a reduction in the initial cost can be achieved.

In carbon steel coated rebars the coating thickness and its quality (integrity) are crucial to guarantee the effectiveness of the protection [94,95]. In galvanized reinforcements, where a protective zinc-based coating is present, having typically a more or less homogeneous pure zinc η-phase on the top, the passive film, produced on the rebar surfaces, can be effective in concrete structures subjected to carbonation induced corrosion or to penetration of chlorides [96,97]. In carbonated concrete the corrosion rate is about 1–2 µm/year, thus the corrosion propagation is very slow [98]. A chloride threshold for pitting corrosion initiation in the range of 1–1.7% by weight of cement has been found, reaching also higher values than these ones, when the coating is constituted by different zinc alloys, which can be obtained from different baths in the process of hot-dip galvanizing of carbon steel reinforcements [99]. Therefore, the chlorides threshold to initiate the pitting corrosion of galvanized steel reinforcements is significantly higher than that generally considered for carbon steel rebars (0.4–1%); hence, advantages can be obtained in terms of service life extension. Furthermore, in the presence of coating discontinuities, owing to bending of rebars or welding operations, which leave uncoated substrate spots, the zinc-based coating determines a cathodic protection of the steel in correspondence of these spots [100].

In epoxy coated rebars, the epoxy resin can provide a barrier protection. This kind of resin is suitable for use in concrete (good resistance to alkaline solution, good mechanical properties, good adhesion to steel and concrete). In the presence of defects, when concrete is carbonated or in the presence of chlorides with a content higher than the chloride threshold, corrosion can occur. In carbonated concrete the attacks are, generally, limited to the area of the defects, thus also the consequences are limited [101]. In chloride contaminated concrete no advantage in pitting corrosion initiation can be achieved in the presence of defects [102]. Moreover, with these bars, the use of electrochemical techniques to assess the corrosion behavior of the reinforcements is not possible due to the presence of the electrical insulating coating. After cutting or welding, or in the presence of defects in the coating, the areas without protection have to be repaired with a paint.

In stainless steel reinforcement the corrosion resistance is given by their chemical composition (alloy elements: mainly chromium, molybdenum, nickel, nitrogen). These rebars, if properly selected, can guarantee also long service lives in harsh environmental conditions without maintenance thanks to their high resistance to corrosion. These steels do not suffer corrosion in carbonated concrete and can resist to chloride induced corrosion also in the presence of very high chloride content (also higher than 5% by cement weight) [103,104,105,106]. For this reason, their use is generally considered in chloride-rich environments with high aggressiveness. To select the most suitable type of stainless steel in terms of corrosion resistance and cost, among the different types available, the chloride threshold for pitting corrosion initiation must be known. In order to limit the costs, stainless steel reinforcement is often used in the most critical parts of the structure (or in a repaired area) and connected with the carbon steel rebars. This coupling does not lead to risk of galvanic corrosion [107].

The use of FRP reinforcement, generally GFRP (Glass Fiber Reinforced Polymers), is still to be considered in the experimental phase. Long-term data on their behavior under different exposure conditions are not available. Despite the fact they do not suffer electrochemical corrosion as steel does, they are subjected to other deterioration phenomena, e.g., due to concrete alkalinity, temperature, and humidity [108].

## 6. Durability Design

Worldwide, corrosion of embedded steel is the main form of premature damage of reinforced concrete structures, hence there is the need to prevent it since the design stage [33,109,110,111,112,113,114]. At this aim several approaches are available that are characterized by different levels of approximation. As introduced for the structural design in the “Model Code for Concrete Structures” issued by the International Federation for Structural Concrete (*fib*) in 2010, a level of approximation is a design strategy where the accuracy of the prevision can be, if necessary, progressively refined through a better estimation of the parameters related to the considered phenomenon [115]. A low level of approximation should be reserved for structures where high accuracy is not required or for a pre-design; conversely higher levels of approximation can be used in cases where higher accuracy is required and it is expected that the solution is closer to the actual behavior.

Dealing with durability, a low level of approximation can correspond to the prescriptive approach, that needs the fulfillments of minimum requirements, whilst through a performance-based approach, which consists of a real durability design, the accuracy of the prevision can be increased.

The prescriptive approach is based on the definition of an exposure class, that describes the aggressiveness of the environment to which concrete will be exposed during its service life, and the subsequent prescriptions regarding the maximum water/cement (*w/c*) ratio and the minimum cement content, according to the EN 206 [116]. These should be associated with minimum values of the concrete cover thickness (related to protection of rebars from corrosion), according to the Eurocode 2 [117]. These simple recommendations apply to any type of cement of the EN 197-1 standard [118] and refer to an intended service life of about 50 years. The prescribed values revealed to be inadequate in some parts of the structures, as those highly exposed to chlorides, e.g., the joints or the splash zone in marine structures [113]. Moreover, it is implicitly assumed that durability performances of concretes made with different types of cement are comparable, whilst it is well known that they behave even significantly different in relation to the resistance to aggressive agents [119,120,121,122,123,124,125]. Finally, this kind of approach does not allow to take into account the advantages of additional protections.

**Figure 2:**
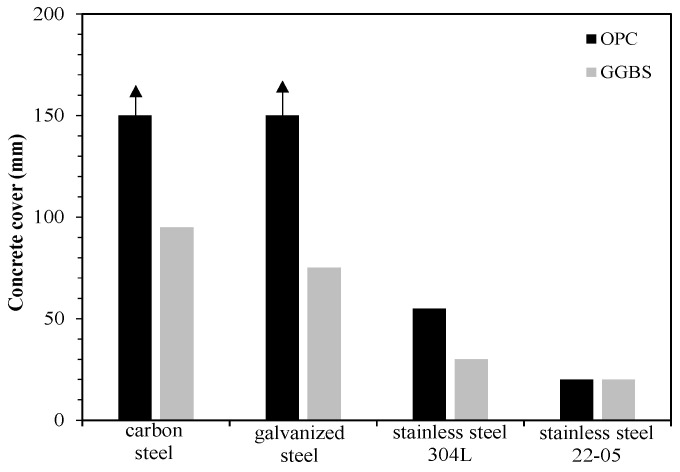
Average value of the concrete cover thickness as a function of the type of concrete (OPC = Portland cement; GGBS = ground granulated blast furnace slag; water/binder = 0.45) and the type of bar that guarantees a service life of 100 years in the splash zone, assuming a target probability of failure of 10%.

The performance-based approach allows to specifically design each structural element in a way that it can withstand the actual local conditions of exposure during the required service life. Among the models proposed in the recent years, the *fib* ‘‘Model Code for Service Life Design’’, published in 2006 [126], is one of the most used. This includes a probabilistic performance-based approach that, modelling the environmental effects on the structure, allows the evaluation of the probability that a pre-defined limit state, which corresponds to an undesired event (e.g., initiation of corrosion, cracking or spalling of concrete cover), occurs. Through these models, different design combinations, together with their reliability, can be compared, as well as the benefits connected with the use of preventative techniques [93,127]. As an example, Figure 2 shows the durability design, carried out through the *fib* Model Code, of a RC element exposed to the splash zone, considering a service life of 100 years and different design options, in term of types of concrete and reinforcement and concrete cover thickness. Their widespread use, however, is still limited, since indications on same input design parameters are lacking and their estimation is entrusted to the experience of the designer. Moreover, since these models are quite young compared to the length of usual service lives of RC structures and feedback data are not available yet, the reliability of their output is still under investigation [128,129].

## 7. Waterproofing Recycled Aggregates

Cement composites can be considered unsaturated porous materials that, when in direct contact with water, are permeated through various transport mechanisms (capillary rise, permeation, diffusion). These processes dramatically affect the durability of the concrete structures since they: (a) expose them to freeze and thaw deterioration, (b) alter cement paste composition/microstructure by dissolution and removal of its ionic compounds, (c) promote the ingress of aggressive ionic agents such as sulfates and/or chlorides.

An important characteristic of a porous material is the capillary water absorption expressed with the absorption coefficient *S* (kg/(m^2^·s^0.5^):

(1) $$S=\sqrt{\left(\frac{\sigma \;cos\vartheta \;r}{2\mu }\right)}\;$$

where *r* is the mean radius of the capillary pore, *σ* the surface tension of the liquid, *ϑ* the water contact angle. Thus *S* is higher as the pore size increases and as the contact angle decreases, so when dealing with porous and hydrophilic materials [130]. The cementitious matrix is made of hydrated products (mainly composed by Ca, Si, Fe) and the aggregates (65–75% of the total volume) are generally natural siliceous or limestone sand and gravel. All of these constituents contribute to the pronounced hydrophilic character of the whole cement composite which is characterized also by a peculiar porosity [131]. On the contrary, polymeric materials are intrinsically hydrophobic as they are rich in low energy groups, e.g., the -CHx ones [132,133]. For these reasons, hydrophobic cementitious materials can be easily obtained by using polymeric aggregates as a partial substitution for natural stones [134].

Replacing natural sand in cementitious mortars with grains of end-of-life tyre rubber, containing isoprene/butadiene chains, strongly reduces the penetration of water drops both in sound and cracked materials. In fact, the hydrophobic character of the aggregates is dispersed in the whole mass of the composite and exerts its effect both on the surface and in the bulk [135]. Fast water absorption, instead, has been detected when inorganic (siliceous) recycled aggregates, such as porous waste glass, have been tested [136].

**Figure 3:**
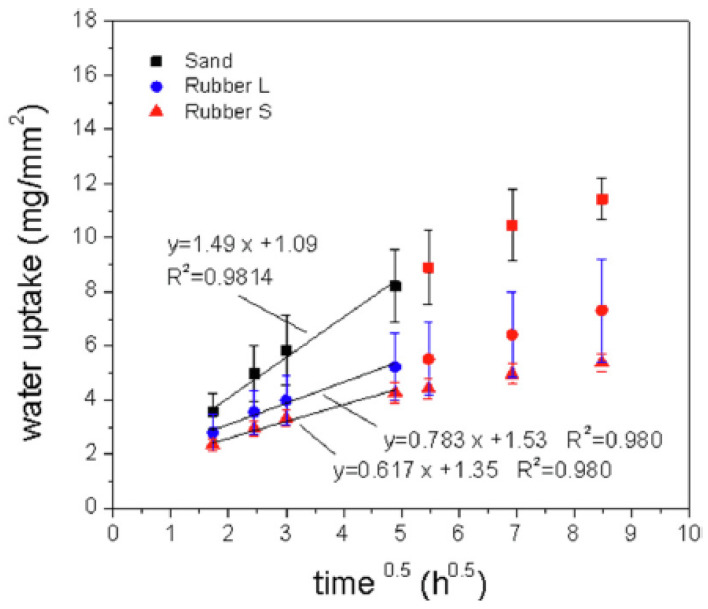
Water uptake as a function of the square root of time for mortars containing siliceous sand (Sand) and rubber grains from end-of-life tires (Rubber). Larger (L) and smaller (S) granulometric fractions of rubber have been separately use.

However, the different surface energy of polymeric aggregates with respect to traditional concrete constituents determines a reduction in aggregate-cement bond, thus increasing both the porosity of the material and the parameter *r* reported in Equation (1). Nevertheless, the effectiveness of these aggregates in hindering the water ingress has been proved in terms of both water absorption rate of microliter water drops [135] and water capillary rise in partially immersed samples [137]. The first method allows a highly space resolved wetting analysis, the latter allows a quick and overall evaluation of the absorption coefficient *S* (Equation (1)). From Figure 3 it is evident that the absorption coefficient (calculated from the slopes of the linear fit) of rubberized mortars is less than a half of those made with natural sand [136]. Furthermore, when exposed to accelerated chloride penetration, a lower corrosion degree of steel reinforcement has been measured in rubberized concrete with respect to ordinary concrete, indirectly confirming the high water resistance of mixtures containing end-of-life tire aggregates [138].

## 8. Durability of Special Mixtures

### 8.1. Fly Ash-Based Geopolymers

A new class of materials known as geopolymers, which are part of the broad class of inorganic matrices named alkali-activated materials (AAM) has rapidly grown in interest in the last two decades in order to reduce the CO_2_ emissions for cement and ceramic materials productions. This new class of materials is based on alkali activation of low calcium aluminosilicate precursors able to consolidate at room or slightly higher temperatures. One of the main advantages of AAM and geopolymers is the possibility to use waste-based powders as for example coal fly ashes derived from coal fired power stations, thus promoting a circular economy approach. Many aspects of geopolymers have been studied, from the synthesis and optimization of aluminosilicate precursors to the properties of the developed products (physical, mechanical, and microstructural performances) [139,140,141,142,143,144,145,146].

As far as geopolymers durability is concerned, interesting results have been obtained about their resistance to sulfate attack [147] and alkali–silica reactions and about the high stability in the presence of fire or freeze–thaw cycles, besides a high adhesion to steel reinforcement [148,149,150,151,152,153], which suggests their use as binder in mortar and/or concrete, or for strengthening applications of reinforced concrete structures [144,154,155,156,157]. If properly designed, geopolymers perform better than ordinary Portland cement when exposed to high temperature. The rapid dehydration of the weakly bound water in the gel does not cause significant damage to the binding structure, therefore mechanical strength is retained and considerable dimensional stability at high temperature is verified [158,159,160,161,162,163]. Recycled refractory particles (RRP) have been used to develop AAM and geopolymers with enhanced performance at high temperatures [164]. RRP do not hinder the alkali activation process, and they reduce heat-induced cracking, increase the maximum temperature of dimensional stability of the composites up to 1240 °C, and improve the linear dimensional stability during heating. In addition, the room temperature curing generates a product less prone to cracking than heat curing when exposed to high temperature [38]. Considering the good performance of AAM as fire resistant materials [165,166,167], research has also focused on the use of lightweight AAM for steel protection (passive fire protection systems) [168,169,170]. Heat-induced cracking in fly ash-based alkali-activated pastes and lightweight mortars was analyzed by in situ acoustic emission detection during complete heating–cooling cycles [171]. Cracking during heating was limited and associated exclusively with the dehydration of the materials. However, samples heated to temperatures above 600 °C exhibited intense cracking on cooling.

The addition of sodium silicate to sodium hydroxide stimulated network formation in geopolymers leading to improved mechanical strength, lowering chloride ion mobility and slightly improving corrosion performances [172,173]. Studies on the corrosion behavior of steel in different room temperature cured alkali-activated fly ash mortars exposed to chloride solution showed that the most compact alkali-activated mortars have higher porosity and lower mechanical properties than a cement-based mortar, but the protectiveness afforded to the rebars is slightly higher than that obtained in traditional mortars [174].

### 8.2. Alkali Activated Materials

Alkali-activated materials are a recent family of binders. In the last decades, they received growing attention from academic research institutions as promising candidates in specific civil applications, such as refractory structures and concrete sewers [165,175,176,177]. Nevertheless, related marketplace shows a certain amount of concern on AAM more extensive use due to the unclear performances in terms of properties and durability, lack of extensive track record, product standards, and tailored polymer admixtures. The significant numbers of raw materials and alkaline activators, that can be used to formulate their mix design, deeply affect fundamental properties, such as shrinkage and cracking behavior, workability, development of mechanical strength, and risk of efflorescence [147,178,179,180,181]. The adopted formulations also determine their resistance to chemical, physical, and transports attack modes. Moreover, the level of confidence is also lowered by the evidence that some durability tests, tuned on Portland based products, fail in predictive ability when applied to AAM [181,182,183].

Results published by several researchers, even in the presence of a multitude of evaluation criteria and methods (length and mass changes, residual mechanical properties, ultrasonic pulse velocity, and elastic modulus) and testing conditions (salt concentration, temperature, time of immersion, periodical solution replacing, and former curing regime) clearly corroborate that AAM exhibit a higher resistance to sulfate attack than that of Portland cement (PC) materials [184,185,186,187,188,189]. The rate, severity, and mechanism of external sulfate attack on AAM concrete, that takes place in soil or marine environments, depend on the permeability of the concrete/mortar, the concentration of sulfates in the waterborne solution, on the cation accompanying the sulfate ions, on nature of the selected reactive powders and activator composition. When aluminosilicates are used as reactive powders (fly ash or metakaolin), geopolymerization leads to a N-A-S-H gel that differs from PC hydration products, characterized by the absence of high-calcium phases. This condition prevents the formation, under the sulfate attack, of gypsum or ettringite and results in a good resistance to sulfate attack [188], that is further increased if Na(OH) instead of Na_2_SiO_3_ is used as activator [185]. When Blast Furnace Slag (BFS) or other high calcium containing reactive powders are used, the formation of a hydrotalcite-type phase and of a hydration product, a calcium aluminosilicate hydrate (C-A-S-H) commonly occurs. It is less crystalline and with a lower CaO/SiO_2_ molar ratio than calcium silicate hydrate (C-S-H) produced by PC hydration. When exposed to sulfate attack, its durability strictly depends on cation accompanying the sulfate ions. Bakharev et al. [184], using ASTM C 1012, investigated the behavior of AAM cured for 28 days in a fog room and then immersed in a 50 g/L Na_2_SiO_3_ or 50 g/L MgSO_4_ solution for 12 months. No sign of deterioration was visible after sodium sulfate attack, whereas matrix degradation with formation of gypsum was observed under magnesium sulfate attack. Similar trend was found by Ye et al. [186].

When Na_2_SO_4_, KOH, and NaOH were used as activators traces of ettringite, a little expansion of the samples was visible and traces of ettringite as well as a reduction in the intensity peak of C-A-S-H gel were detected by XRD diffractometry. This behavior was related to limited gel decalcification and dealkalization, whereas, when Na_2_CO_3_ was used, the presence of CO_3_^2−^ suppressed ettringite formation. Under magnesium sulfate attack a more sever degradation process was observed with mechanisms that depend on the activators’ type. When NaOH or sodium silicate were used, firstly brucite was formed. It acted as a protective surface layer by delaying external ion migration, but lowered the pH of the pores solution to 10.5 thus promoting C-A-S-H gel decalcification, but not its dealumination, and gypsum production. The further migration of Mg^2+^ and SO_4_^2−^ and their reaction with decalcified C-A-S-H produced a magnesium-aluminosilicate-hydrate (M-A-S-H) and/or silica gels. The absence of ettringite was ascribed to the unfavorable pH and limited amount leached aluminum. The AAM obtained using Na_2_SO_4_ showed the weakest resistance against MgSO_4_ attack, due to lack of brucite protective layer due to the absence of hydroxide ions.

Ismail et al. [189] also investigated the resistance to sulfate attack of blended AAM, obtained using BFS and fly ash. Again, a key role is provided by the nature of the cation accompanying the sulfate, with negligible degradation after immersion in Na_2_SO_4_ solution and a more severe damage in MgSO_4_ solution, with matrix degradation and loss of samples integrity.

### 8.3. Calcium Sulfoaluminate Cements

Calcium sulfoaluminate (CSA) cements are special hydraulic binders generally produced from limestone, bauxite, and gypsum and they represent an important alternative to Portland cement (PC). Compared to PC, CSA cements exhibit more pronounced environmentally friendly features mainly thanks to lower synthesis temperatures (~1350 °C) and reduced limestone content (~40%) both determining a strong decrease of kiln thermal input and CO_2_ emissions. Furthermore, CSA clinker, which can be produced also by using industrial wastes often difficult to reuse, is more friable than PC clinker and is blended with relatively high amounts of calcium sulfates to produce CSA cements [190,191,192,193].

CSA cements contain C_4_A_3_$ (ye’elimite, the main component), calcium sulfates and a variety of calcium aluminates and silicoaluminates. The most important properties of these binders are regulated by ettringite (C_6_A$_3_H_32_), generated upon hydration of C_4_A_3_$ together with calcium sulfates. Depending on the conditions of C_6_A$_3_H_32_ formation, several technical properties can be attained (e.g., rapid-hardening, good dimensional stability, low permeability and solution alkalinity, or shrinkage compensation/self-stressing behavior) [194,195,196,197,198].

Compared to PC, there are relatively few durability studies on CSA-based cements. These mixtures have proved to be highly resistant to freeze–thaw and chemical attacks promoted by sulfates, chlorides, magnesium, and ammonium salts [193,199,200,201,202,203]. These features are mainly related to the lower porosity developed by CSA cements if compared with PC binders. In fact, porosity measurements on hydrated CSA cements, carried out with mercury intrusion porosimetry, have revealed the presence of pores with threshold radius below 25 nm [204] and only a minor content of larger pores forming an interconnected pore network [193,204], leading to low permeability [201].

As far as the carbonation is concerned, it has been found that it is due to ettringite decomposition into calcite, gypsum, and aluminum hydroxide. By now, the results obtained from carbonation tests are contradictory. In fact, a few studies state that CSA cements carbonate faster than PC [205,206,207]; other papers report that both CSA cements and PC display the same carbonation rate [208] and finally, according to other findings, CSA cements perform better than PC [209].

The behavior of CSA-based binders in relation to the durability of reinforced concrete structures is still not clear. Nevertheless, recent studies have shown that steel bars in CSA reinforced concretes, when put in chloride-free environments, seem to be protected from corrosion, despite that their alkalinity (pH = 11.5–12.0) is lower than that of PC (pH = 12.5–13.5) but sufficient to promote the passivation of embedded steel [210]. Moreover, corrosion tests on carbonated CSA concrete showed negligible corrosion rate of steel in environments up to 95% relative humidity at 20 °C temperature. Furthermore, the low alkalinity is surely favorable towards the alkali aggregate reaction [200,202,203].

## 9. Conclusions

This paper highlights the possible strategies for obtaining sustainable and durable concretes. In particular, it is shown how it is possible to realize durable reinforced concrete structures in different aggressive environments through an appropriate design that starts from a proper concrete composition (binders type and dosage, water content, aggregates, admixtures), passes through the choice of reinforcements (traditional carbon steel, galvanized steel, stainless steel, composite materials or coated reinforcements), and ends with the selection of additional solutions such as inhibitors, coating, self-healing techniques or waterproofing aggregates.

## Data Availability

Not applicable.
